# Sarcoid‑like granulomatous inflammation in a carotid body paraganglioma: A case report and mini‑review of the literature

**DOI:** 10.3892/mi.2023.107

**Published:** 2023-08-30

**Authors:** Ari M. Abdullah, Fahmi H. Kakamad, Soran H. Tahir, Aso S. Muhialdeen, Abdulwahid M. Salih, Hawbash M. Rahim, Bruj Jamil Mohammed, Fakher Abdullah, Dahat A. Hussein, Shvan H. Mohammed

**Affiliations:** 1 Sulaimani Teaching Hospital, Sulaimani, Kurdistan 46001, Iraq; 2 Department of Scientific Affairs, Smart Health Tower, Sulaimani, Kurdistan 46001, Iraq; 3 Kscien Organization for Scientific Research, Sulaimani, Kurdistan 46001, Iraq; 4 College of Medicine, University of Sulaimani, Sulaimani, Kurdistan 46001, Iraq; 5 Department of Medical Biology, Faculty of Medicine, Tokat Gaziosmanpaşa University, Tokat 60000, Turkey; 6 Shar Hospital, Department of Medicine, Sulaimani, Kurdistan 46001, Iraq

**Keywords:** paraganglioma, carotid body tumor, carotid body paraganglioma, sarcoid-like granulomatous inflammation, sarcoid-like reaction

## Abstract

Sarcoid-like granulomatous inflammation (SLGI) is defined as the development of non-necrotizing epithelioid granulomas in patients who do not meet the criteria for systemic sarcoidosis. Its occurrence is known to be linked to diverse conditions, including malignancies, infections, the use of certain drugs and inorganic substances. To the best of our knowledge, the available literature to date lacks any description regarding SLGI in a paraganglioma. The present study describes the first case of SLGI in a carotid body paraganglioma (CBP). A 54-year-old female patient presented with anterior neck swelling for 27 years without any other symptoms. An ultrasonography revealed a multinodular goiter with retrosternal extension and a solid lesion on the right side of the neck measuring 40x30x22 mm, which was suggestive of a CBP. The patient underwent a total thyroidectomy, and the right-side cervical mass was resected via another longitudinal incision. A histopathological examination of the thyroid specimen revealed findings of a multinodular goiter. Sections from the right-side cervical mass confirmed the diagnosis of CBP accompanied by multiple, well-formed, small-medium sized, non-necrotizing epithelioid granulomas associated with multinucleated giant cells, indicative of SLGI. Non-necrotizing epithelioid cell granulomas (as with SLGIs), identical to those observed in sarcoidosis, may rarely be observed in patients who do not meet the criteria of systemic sarcoidosis; however, they have been observed in association with various neoplasms. It is thus crucial to distinguish them from actual sarcoidosis, as misdiagnoses may lead to severe consequences. The presence of SLGIs accompanying a paraganglioma is an extremely rare phenomenon. Due to this, it is difficult to conclude if it confers a better prognosis or not.

## Introduction

Paraganglioma is a somewhat rare neuroendocrine tumor that most often arises from the paraganglion tissues of the adrenal glands. Only in a tenth of cases, extra-adrenal localization may occur, of which 85% reside in the abdomen, and only 3% occur in the head and neck region (1). Usually, these tumors are considered benign; however, in ~19% of instances, malignant transformation may occur (2). In the adrenal glands and abdomen, paraganglioma arises from sympathetic paraganglia. On the other hand, parasympathetic paraganglioma is almost exclusive to the head and neck area (3). According to the anatomic site, the tumor is referred to as vagal paraganglioma, carotid body paraganglioma (CBP) (or a carotid body tumor), jugular paraganglioma and tympanicum paraganglioma (1).

One of the chronic inflammation patterns that occurs when the complete clearance of antigenic stimuli is not achieved by the cellular immune system, is granulomatous inflammation, such as that observed in sarcoidosis (4). Non-necrotizing epithelioid cell granulomas, such as those observed in sarcoidosis may rarely be observed in patients who do not meet the criteria of systemic sarcoidosis (5). This phenomenon is known as sarcoid-like granulomatous inflammation (SLGI), and it can be difficult to distinguish between this and systemic sarcoidosis (6,7).

It is crucial to distinguish SLGIs from actual sarcoidosis, as misdiagnosis may lead to severe consequences (8). Such phenomena have been observed in association with various neoplasms, including renal cell carcinoma, breast, parathyroid, germ cell, gastrointestinal and salivary gland tumors (5-7,9-12). However, to the best of our knowledge, the available literature to date lacks any description regarding such a reaction in a paraganglionic tumor.

The present study describes a case with the first reported association of a CBP with SLGI in the absence of any criteria of systemic sarcoidosis or other SGLI-associated conditions.

## Case report

### Patient information

A 54-year-old female patient visited Smart Health Tower (Sulaimani, Iraq) with the presentation of anterior neck swelling for a period of 27 years without any other associated symptoms. She was a married housewife and did not have a history of smoking.

### Clinical findings

Upon examination of the swelling and upon inspection, a grade 3 multinodular goiter (MNG) was suspected as the patient had a thick neck mass visible from a distance of 5 meters, and by palpation, the enlargement was noted to be firm. In addition, a right-side lateral neck mass was observed, which was firm on palpation and movable side to side, but not vertically.

### Diagnostic approach

Routine laboratory investigations mostly revealed normal findings. However, the patient had decreased thyroid stimulating hormone levels (<0.4 mIU/l), indicating toxic MNG. An ultrasonography revealed MNG with retrosternal extension and a solid lesion on the right side of the neck measuring 40x30x22 mm, which was suggestive of a CBP. Further imaging diagnosis via a computed tomography scan also revealed the presence of MNG in both thyroid lobes, with the largest lobes measuring 75x47 and 87x45 mm on the right and left side of the thyroid, respectively, and a lateral solid hypervascular mass between the internal and external carotid arteries (data not shown).

### Therapeutic intervention

Under a general anesthesia, in a supine position, and via a collar incision, a total thyroidectomy was performed for the patient while preserving the parathyroid gland, and the other cervical neck mass was also resected via another longitudinal incision. A histopathological examination was performed for both masses. Sections from the thyroid gland revealed multiple nodules of different sizes composed of hyperplastic thyroid follicles arranged as large follicles, filled with colloid material, and associated with cystic changes, hemorrhage, fibrosis, cholesterol cleft formation and calcifications, indicating MNG (data not shown).

**Figure 1:**
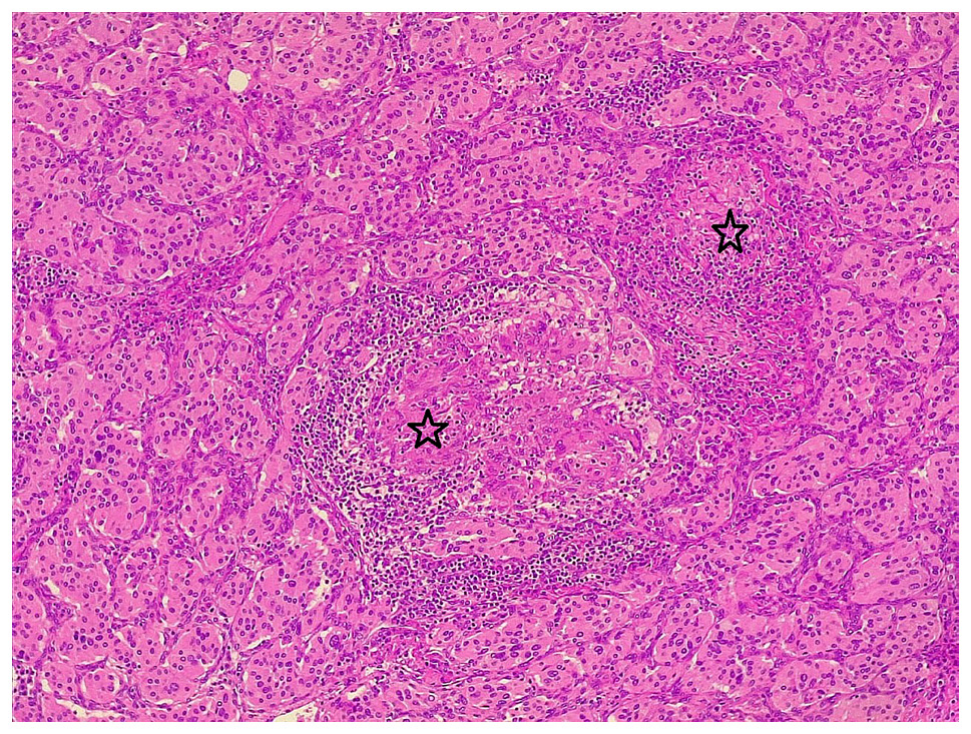
The section (hematoxylin and eosin staining) illustrates nests and sheets of round-polygonal cells with an abundant eosinophilic cytoplasm in a hyalinized vascular stroma with well-formed non-necrotizing epithelioid granuloma (stars) and multinucleated giant cells.

For histopathological analysis, the sections (4-µm-thick) were paraffin-embedded and fixed with 10% neutral buffered formalin at room temperature for 24 h. The sections were then stained with hematoxylin and eosin (Bio Optica Co.) for 1-2 min at room temperature. They were then examined under a light microscope (Leica Microsystems GmbH). Sections from the right-side cervical mass revealed a well-demarcated mass composed of nests and sheets of round-polygonal cells with abundant eosinophilic granular cytoplasm in a hyalinized vascular stroma, focal areas of atypia and focal heavy lymphoplasmacytic infiltration, with the presence of multiple, well-formed, small-medium size, non-necrotizing epithelioid granulomas associated with multinucleated giant cells, confirming the diagnosis of CBP with SLGI and focal lymphoplasmacytic infiltration (Fig. 1).

### Follow-up and outcome

The surgery was uneventful, and the patient was discharged in good health. Following a follow-up period of 6 months by ultrasound, the patient was in good health without any sign of recurrence.

## Discussion

Non-necrotizing epithelioid cell granuloma, identical to that observed in systemic sarcoidosis, is a rare, yet well-reported phenomenon found in association with various primary neoplasms, infections, drugs (antibiotics, methotrexate, and non-steroidal anti-inflammatory drugs) and inorganic substances (11). When this occurs, it is termed ‘SLGI, sarcoid-like reaction, sarcoid-like granulomatosis, or sarcoid-like granuloma’ (5-7,9). The present study describes a previously unpublished association of CBP and SLGI without any relevant systemic conditions.

Paragangliomas are a family of sympathetic and parasympathetic paraganglionic tumors. The sympathetic types originate from within the abdomen and adrenal glands, which are referred to as abdominal paraganglioma and pheochromocytomas, respectively. By contrast, the parasympathetic types mainly arise from the head and neck region (3,13).

The carotid body is a chemoreceptor organ that is highly vascularized (also reflected in their imaging appearance), reddish brown in color, and located within the adventitia posteromedial to the common carotid artery bifurcation. Its role involves the autonomic regulation of temperature, cardiovascular, respiratory systems, and acute adaptation to fluctuating concentrations of oxygen, carbon dioxide and pH. It consists of sustentacular cells and chief cells derived from the neuroectoderm and neural crest cells, respectively. Tumors originating from the chief paraganglia cells are termed CBP (3). Even though it is the most common type of head and neck paraganglioma, it is still an overall rare neoplasm that requires a thorough examination for appropriate diagnosis and management. The tumor can be familial or sporadic, with the latter being more common (14).

CBPs have been reported to be more prevalent among females and are most often observed in the third to the sixth decade of life. The neoplasm usually presents as a non-tender, slow-growing mass, and rarely does the tumor demonstrate a bruit/thrill or transmit a carotid pulse. The tumor may encase the external and internal carotid arteries as it increases in size, although it does not narrow them. Even though CBP is often asymptomatic in the initial phase, due to its proximity to the adjoining vessels and nerves, symptoms may manifest with an increase in size, such as a hoarseness of voice, odynophagia, dysphagia and other cranial nerve deficits. Hypertension, sweating and headaches may also be observed in some patients as a result of the secretion of vasoactive catecholamines by the CBP (3,15). These descriptions are in accordance with the case described herein along with our case, as the patient was a 54-year-old asymptomatic female. In patients with multiple occurrences, an age <45 years, bilateralism, a previous history or a current history of CBP, and a positive family history, performing genetic testing and biochemical phenotype assay are recommended to aid in the early detection of complications and the initiation of proper intervention therapies. Conducting appropriate family screening may also aid in improving the overall prognosis (14).

Upon a histological examination, CBPs have a characteristic growth pattern known as ‘zellballen’, an organoid or well-developed nested growth pattern of the tumor cells with a stromal component between fragile fibrovascular tissue and supporting sustentacular cells along the edges of the zellballen or cell nests. The tumor cells are mostly chief cells with dispersed chromatin, hyperchromatic, round nuclei and an abundant granular cytoplasm that may be either basophilic to eosinophilic in color (12,15). Although their vascularity makes the condition challenging, surgical removal is the recommended management approach associated with a good prognosis. In cases of malignancy and metastatic disease, radiation and chemotherapy do not appear to be greatly beneficial (15).

A number of granulomatous conditions are able to mimic sarcoidosis, clinically and histologically, including sarcoid-like granulomatous reactions, infectious granulomatous diseases, granulomatous drug reactions, neoplastic disorders, immunodeficiencies and systemic disorders with granulomatous characteristics (Rosai-Dorfman disease and Blau syndrome) (8,16). Distinguishing SLGIs from actual sarcoidosis requires a careful review of a patient's complaints, demographics, medical history, clinical examination, laboratory findings, imaging and histological features. Some clinical characteristics may immediately eliminate the diagnosis of sarcoidosis due to atypical characteristics, such as an age <25 years or >45 years, a high fever, acute or subacute dyspnea and hemoptysis (16).

The first case of SLGI was reported by Wolbach in 1911(17). Since then, this reaction has been reported in association with various neoplasms. Some studies have aimed to determine the frequency of these reactions in different neoplasms, with the reported frequency ranging from 4% in carcinomas to 20% in lymphomas (5,12). Sarcoid-like reactions often occur in organs that dendritic cells can reach, although rarely at the tumor site (6).

In patients with no history of sarcoid disease, non-necrotizing epithelioid cell granulomas may rarely occur within tumors. The pathogenesis of SLGIs remains to be fully established. It has been reported that it may occur as the result of soluble tumor antigens or cancer-related antigenic factors being shed into the blood, leading to immunological hypersensitivity and granulomatous inflammation (7).

Histologically, SLGIs are similar to sarcoidosis granulomas. They are comprised of well-defined non-caseating granulomas with the focal accumulation of multinucleated giant cells and epithelioid cells (5,9). Usually, no necrosis is observed, although a few patients may be present with fibrinoid necrosis (12). A similar observation was made in the case described herein.

The presence of SLGI is an indicator of an immunological response of macrophages by activated T-lymphocytes to a neoplasm. Even though the presence of T-lymphocytes should translate to a protective role and better prognosis, currently, the prognostic value of SLGIs is not yet well understood (9). It has been reported to be associated with a good prognosis in various lesions, including gastric cancers and Hodgkin's disease (12). However, some studies have reported no prognostic significance in lung cancers (18,19). In a previous study, in a case of renal cell carcinoma and SLGI, the patient succumbed due to metastasis 6 months following ta nephrectomy; however, the authors argued that the patient's death was more likely influenced by sarcomatoid features (20). Although the case described in the present study is of interest, the authors' were not able to evaluate prognostic significance due to a limited follow-up period and the fact that the case was the first of its kind, at least to the best of our knowledge. All the referenced studies in this report were evaluated for their credibility before being cited (21).

In conclusion, SLGIs accompanying a paraganglioma is an extremely rare event that has not previously been reported, at least to the best of our knowledge. The case described in the present study is the first to be reported; there was no evidence of systemic sarcoidosis or tuberculosis. Due to its rarity, it is difficult to conclude if it confers a better prognosis or not.

## Data Availability

The datasets used and/or analyzed during the current study are available from the corresponding author on reasonable request.
